# Effects of Mind-Body Training as a Mental Health Therapy in Adults with Diabetes Mellitus Type II: A Systematic Review

**DOI:** 10.3390/jcm12030853

**Published:** 2023-01-20

**Authors:** Beatriz Ruiz-Ariza, Fidel Hita-Contreras, Carlos Rodríguez-López, Yulieth Rivas-Campo, Agustín Aibar-Almazán, María del Carmen Carcelén-Fraile, Yolanda Castellote-Caballero, Diego Fernando Afanador-Restrepo

**Affiliations:** 1 Department of Health Sciences, Faculty of Health Sciences, University of Jaén, 23071 Jaén, Spain; 2 Lecturer University Schools Gimbernat, University of Cantabria, 39005 Santander, Spain; 3 Faculty of Human and Social Sciences, University of San Buenaventura—Cali, Santiago de Cali 760016, Colombia; 4 Faculty of Health Sciences and Sport, University Foundation of the Área Andina—Pereira, Pereira 660004, Colombia

**Keywords:** diabetes mellitus, yoga, mindfulness, depression, anxiety, stress, psychological

## Abstract

The increase in the prevalence and disease burden of diabetes has highlighted the need to strengthen a comprehensive care system that includes mental health treatment. A systematic review was carried out to analyze the effectiveness of mind-body training as a therapy for the mental health management of adult patients with type 2 diabetes mellitus (T2DM) following the PRISMA 2020 guidelines. Pubmed, Scopus and Web of Science databases were consulted between November and December 2022. Eight articles were selected according to the inclusion and exclusion criteria. Only randomized controlled trials were included. The interventions focused on mindfulness and yoga with variable durations of between 8 weeks and 6 months. Four of the included studies observed statistically significant changes (*p* < 0.05) in anxiety. Six articles determined that mind-body training was effective for treating depression. Finally, five articles found favorable effects on stress, while one did not observe changes at 8 weeks of intervention or after 1 year of follow-up. The evidence supports the use of mind-body training to reduce stress, depression, and anxiety levels in the adult population with T2DM, which makes this type of training a valuable intervention to be included in an integral approach to diabetic pathology.

## 1. Introduction

Type 2 diabetes mellitus (T2DM) is caused by the inefficient use of insulin by the body and in more than 95% of cases is largely due to obesity and lack of physical exercise [1]. The worldwide prevalence of T2DM has almost doubled since 1980 (from 4.7% to 8.5%) in the adult population [2].

The burden of disease and the demand for care related to T2DM is increasing, therefore the need to strengthen a comprehensive care system that includes different approaches to care, such as mental health, has become evident [2]. Several forms of emotional stress are associated with an increased risk of developing T2DM. These are depression, general emotional stress, anxiety, anger/hostility, and sleep problems [3]. The evidence suggests that depression is from two to three times more common in people with diabetic pathology than in those without [4]. Likewise, other studies have shown that patients with diabetes are twice as likely to suffer from anxiety and depression than the general population [5,6].

The literature supports the link between mental health impairment, the utilization of health resources, and lost productivity in people with diabetes. It also highlights the need for urgency in the detection and treatment of depression as part of the care process and the public health initiative to improve the health outcomes of people with this diagnosis [6], since a change in mental health, such as depressive episodes, represents a risk in the conventional treatment of T2DM [7].

Among the complementary therapy options for pharmacological treatment is physical exercise [8], since it is a useful way to address physical health by counteracting risk factors such as obesity and a sedentary lifestyle, which have an effect on the control of body mass index (BMI), waist circumference and systemic blood pressure [9,10]. Likewise, structured physical exercise brings benefits to mental health [11], significantly reducing depressive symptoms, lowering anxiety levels, and improving sleep patterns and well-being [12,13].

Mind-body training focuses on achieving a high degree of awareness of the body, through breathing exercises and following external instructions that allow the integration of the physical and mental components [14]. The main feature of the training is movement-based mindfulness. It was designed to focus on body sensations, promote relaxation and release negative emotions [15].

Among the most recognized mind-body training methods are Pilates, yoga, and mindfulness. These methods have gained relevance as some of the interventions offered to the population with diabetes, since they have been reported to cause improvements in postprandial glycemia, fasting blood glucose, glycosylated hemoglobin, triglycerides, total cholesterol and low-density lipoproteins in diabetic patients [16,17,18]. Therefore, this review aims to ascertain the mental benefits of this type of training in patients with T2DM.

## 2. Materials and Methods

This systematic review aims to evaluate the effectiveness of mind-body training as a therapy for addressing the mental health of adult patients with T2DM. The review was carried out in compliance with the guidelines established in the PRISMA 2020 document [19] and the pre-specified protocol registered in PROSPERO (CRD42022384243). Likewise, the methodological recommendations of the “Cochrane Manual for the Elaboration of Systematic Reviews of Interventions” [20] were followed.

### 2.1. Information Sources

The literature search was conducted between November and December 2022 in the Pubmed, Scopus and Web of Science databases.

### 2.2. Search Strategy

Different keywords were used, connected in the following search string: (“Diabetes Mellitus”) AND (“Pilates” OR “Yoga” OR “Tai Chi” OR “Core-Based” OR “Mind-Body”) AND (“Depression” OR “Anxiety” OR “psychological distress” OR “mental health” OR “happiness” OR “Wellness”).

### 2.3. Inclusion Criteria

The articles included had to meet the following inclusion criteria: (i) studies referring to mind-body training in people diagnosed with T2DM; (ii) randomized controlled clinical trials; and (iii) studies focused on adults.

### 2.4. Exclusion Criteria

Studies that did not measure anxiety, depression and/or stress were excluded. Likewise, studies focused on populations with type I diabetes mellitus, renal failure, acute infections, neurological diseases, and hormonal disorders, as well as those in which the patients had a history of altered cognitive or mental functions were excluded. Finally, any type of gray literature publication, books or divulgation articles were also excluded.

### 2.5. Studies Selection Process

The article selection was carried out using the Rayyan virtual tool [21] (https://rayyan.qcri.org/welcome accessed on 19 December 2022). Using Rayyan, the articles were filtered and duplicates were eliminated; subsequently, the title and abstract were screened, selecting the articles that met the inclusion criteria. Two of the authors, blinded, evaluated each article; in cases of conflicting decisions, a third author defined the inclusion or exclusion of the article.

### 2.6. Data Extraction

The main variables of the review focused on measuring mental health outcomes: depression, anxiety, and stress. Data on authors, year of publication, country, population characteristics, intervention characteristics (frequency, intensity and duration), as well as the activity of the control group, follow-up time, and results were included.

### 2.7. Methodological Quality Assessment

To evaluate the methodological quality of the articles included in this review, the PEDro scale was used, which is one of the scales with the highest reliability index for the evaluation of the methodological quality of the different publications [22]. This scale is composed of eleven items which evaluate the internal and external validity and statistical support of the publication [23]. The first item is the only one that comprises external validity and is not included in the final sum. Each of the remaining items is scored from zero to one, according to whether it appears (one) or not (zero) in the article. The scale has a maximum score of 10, where only the rating of items 2–11 is considered.

Articles with a score lower than four points will be classified as “poor”, from four to five points as “fair”, between six and eight points as “good” and between nine and ten points as “excellent” [24].

## 3. Results

### 3.1. Selection of the Studies

**Figure 1:**
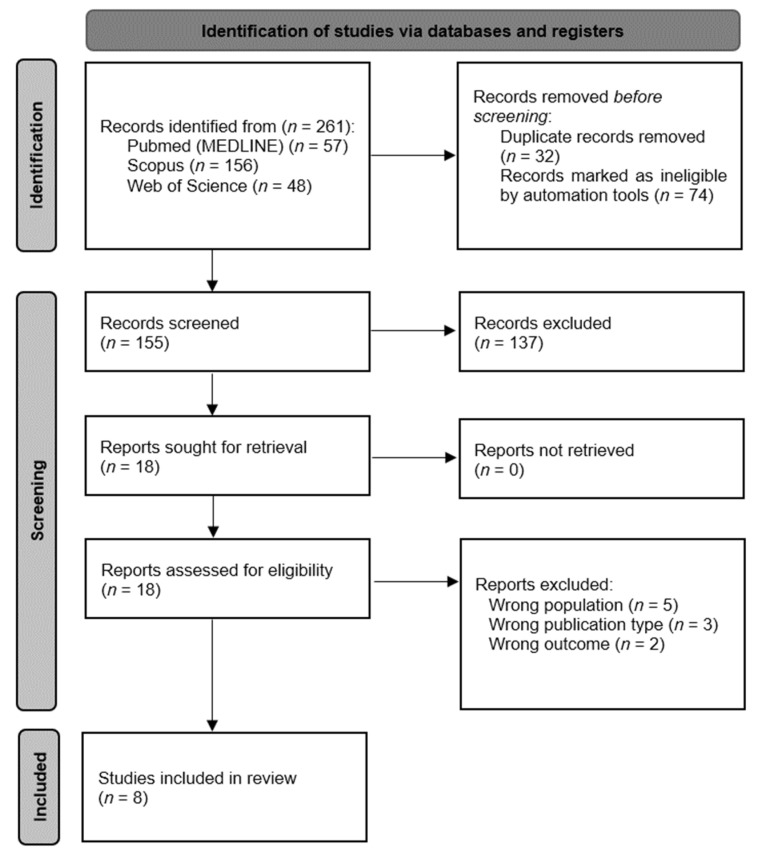
Flow diagram of the study selection process.

The initial search of the databases yielded a total of 261 studies, from which 32 were removed as duplicates and 74 were marked as ineligible by automation tools when applying the randomized controlled clinical trial and article filters. This first process left 155 articles that were screened by two independent authors, excluding 137 articles. A total of 18 articles were sought for retrieval and full-text evaluation, leaving eight articles [25,26,27,28,29,30,31,32] that met all eligibility criteria (Figure 1).

### 3.2. Methodological Quality

Methodological quality was assessed using the PEDro scale. The scores of two of the included articles [27,29] were found on the PEDro website, while the other six were calculated manually [25,26,28,30,31,32]. None of the included articles had poor methodological quality, three had fair quality [25,28,31], while the remaining five had good methodological quality [26,27,29,30,32]. The main weakness in methodological quality was that none of the studies succeeded in blinding the participants or the therapists. Additionally, the study by Sarika et al. [28] was the only one that did not present a baseline comparison (Table 1).

### 3.3. Characteristics of the Studies

Only randomized controlled trials were included in this systematic review. The included studies were published in the United States [25,29,31,32], Greece [26], United Kingdom [27], India [28] and The Netherlands [30], however, the studies were conducted in Germany [25], Greece [26], Taiwan [27], India [28,29,32], Iran [30], and China [31].

A total of 835 adults with an average age of 59.57 ± 7.66 who had T2DM were selected to participate in the included studies. A total of 423 were assigned to a mind-body training intervention group, while the rest of the participants were assigned to a control group (Table 2).

### 3.4. Characteristics of Study Interventions

Three articles [25,27,30] used mindfulness techniques as an intervention, four [26,28,29,32] used yoga and one [31] combined both interventions. In the case of the mindfulness-based interventions, the interventions lasted between 8 [25], 9 [27], and 12 weeks [30], with an approximate volume of 1.5 h, a frequency of 1 session per week [25,27,30] and were all performed in groups and under the guidance of a specialized instructor. In contrast, the duration of the yoga-based interventions varied between 8 weeks [26], 3 months [28,29] and 6 months [32], where participants performed short sessions of about 25–30 min, with a variable frequency of 1/day [28,29], 2/day [26] or 1/week [32], independently with support material [26,28,29] or with direct guidance from an instructor [32]. Finally, the intervention of Chen et al. [31], where mindfulness was combined with yoga, lasted 8 weeks and every 2 weeks, through educational material mediated by platforms, the exercises varied between mindful breathing, body scanning, mindful yoga, and mindfulness meditation.

Only two of the included studies reported adherence to treatment. Singh et al. [29] reported an adherence of 83% to their yoga-based intervention, while Yadav et al. [32] assured a 100% adherence. The other studies that performed interventions that included patients performing their exercises at home and independently [26,28,31] established the use of a diary where the participants recorded both the sessions completed and not completed, as well as the reason why they could not perform them.

Regarding follow-up, only one of the included studies [25] reported a follow-up after the end of the intervention that showed statistically significant changes on the depression variable as evaluated with the Patient Health Questionnaire (PHQ) after 1 year following the intervention.

### 3.5. Anxiety

To assess anxiety, Spielberger’s State-Trait Anxiety Inventory [29], the DASS-21 [30,32] and the SCL-90 [31] were used. Statistically significant changes were observed in all of them, irrespective of the intervention used. Although Chen et al. [27] also applied the DASS-21, they did not report values for anxiety.

### 3.6. Depression

Six of the included studies [25,27,29,30,31,32] in this review evaluated the effects of mind-body interventions on depression. Statistically significant differences in favor of the intervention groups when compared with the control groups were found in all of them. For this variable, different measurement instruments were used, such as the PHQ [25], Depression, Anxiety and Stress Scale-21 (DASS-21) [27,30,32], Symptom Checklist 90 (SCL-90) [31] and the Beck Depression Inventory [29].

### 3.7. Stress

Stress was measured with the PHQ [25], Perceived Stress Scale [26,28], Relocation Stress Scale [27] and DASS-21 [30,32]. The study by Hartmann et al. [25] was the only one that did not find statistically significant changes in stress either at the end of the intervention or after 1 year of follow-up when comparing the intervention group (IG) with the control (CG) (PHQ stress score at 8 weeks, IG: 4.90 ± 0.47/CG: 5.10 ± 0.58, *p* = 0.751) (PHQ stress score at 1 year of follow up, IG: 5.00 ± 0.42/CG: 6.20 ± 0.52, *p* = 0.071).

## 4. Discussion

The aim of this review was to determine the effects of mind-body training on different mental health variables in people with T2DM. Eight randomized controlled clinical trials that met the selection criteria were included [25,26,27,28,29,30,31,32]. All the evidence obtained supports the use of mind-body training as an additional therapy for the management of depression, anxiety, and stress in the study population. However, due to the heterogeneity of the measurement methods employed by the different studies, it was impossible to calculate an effect size for this intervention. Even though three of the studies employed the same measurement instrument (DASS-21), it was applied in different ways by each author. Only Ravari et al. [30] applied the instrument without modifications, while Yadav et al. [32] and Chen et al. [27] modified it, which, combined with the heterogeneity present in the other studies, ultimately made it impossible to perform a meta-analysis.

Mind-body training is a topic that in recent years has attracted the attention of multiple researchers. Different studies have determined the effects of this intervention in patients with chronic obstructive pulmonary disease [33], insomnia [34], chronic pain [35], mild cognitive impairment [36], and fibromyalgia [37], among others. Additionally, in patients with diabetes, it has been observed that mind-body training, such as yoga or mindfulness, improves variables associated with glycemic control (HbA1c, fasting blood glucose and post-prandial glucose), in addition to improving the lipid profile, blood pressure, BMI and cortisol levels [38,39].

Regarding the methodological quality of the included articles, it is important to highlight that none of them was of excellent quality, ranging only between fair and good. The main problem was that none of the studies blinded their patients or therapists, and most of them did not carry out an adequate allocation, which could have altered the results. Non-blinding of patients or therapists and inadequate allocation concealment have been associated with an exaggeration of results of 13% and 7%, respectively [40]. Furthermore, the variety in the intervention protocols in terms of duration, frequency and type prevents the estimation of the dose-response of the mind-body training to obtain positive effects on the evaluated variables and makes it impossible to compare studies in this review.

Mind-body training generated favorable effects on anxiety, depression, and stress in patients with T2DM. The effects of mind-body interventions on anxiety could be attributed to the association between meditation and the acute increase of thalamic GABA levels [41,42], which, by its nature, functions as a pharmacological agent against anxiety [43]. Moreover, evidence related to this type of meditation suggests that it induces an internal relaxation of the autonomic nervous system without leading to lethargy while increasing the activity of the immune system [44,45] and modulating different neurotransmitters such as serotonin and norepinephrine, thus improving different cognitive and psychological aspects, including depression [46].

The systematic review with meta-analysis made by Pascoe et al. [47] suggests that yoga decreases blood pressure, cortisol, and cytokines levels independently of the study population, which would explain the decrease in stress presented by the studies included in this review. Although mind-body training-based interventions have been shown to have effects on stress in different conditions such as neurofibromatosis type 2 [48], mild cognitive impairment, [49] and others [50,51,52], the study by Hartmann et al. [25] included in this review reports the opposite, finding no statistically significant changes between the IG and the CG after the intervention (MI: 4. 90 ± 0.47/CG: 5.10 ± 0.58, *p* = 0.751). However, this is explained by the fact that during the intervention protocol, 9 of the 57 participants attended slightly more than half of the sessions, altering the results.

In coherence with the above-mentioned and highlighting the fact that all studies established a system to follow up the subjects’ compliance with the sessions, either utilizing a diary or in situ interventions, it could be assumed that adherence has a significant role in the effectiveness of mind-body training. Since adherence to interventions like mind-body training is influenced by multiple factors such as teacher competence, reviewing of home assignments, [53] and motivation to change [54], it is important that future research has a correct protocol to follow that mitigates these factors to ensure high compliance.

The results obtained in this review suggest that the completion of the sessions in situ or at home, individually or in groups, does not influence the benefits of mind-body training, this is congruent with the findings of Parsons et al. [55] who, after a systematic review with meta-analysis, support the practice of these types of interventions at home. Finally, in mind-body training, as with any other training, more practice leads to greater benefits.

This review has several limitations. First, the high level of heterogeneity in the instruments used to measure the variables and the various interventions made it impossible to perform a meta-analysis to establish the size of the effect of mind-body training on anxiety, depression, and stress in patients with T2DM. Second, the low methodological quality of the studies could lead to a bias and an overestimation of the results, therefore, they should be interpreted with caution. Third, 75% of the studies were conducted in Asia, so a geographic bias is identifiable, making it impossible to generalize the results to populations in other regions.

## 5. Conclusions

The main interventions based on mind-body training that were implemented with the aim of improving mental health in adults with T2DM were mindfulness and yoga, which demonstrated effectiveness in reducing levels of anxiety, depression and stress despite the variation in the protocols and the variation of instruments applied for the assessment of the outcomes. The effects of mind-body training are mainly conditioned by the adherence to the protocol, while the way it is performed either at home or a specific location does not seem to have any influence. Therefore, it is suggested that clinicians focus more on session compliance than on the modality employed. However, the low methodological quality of the studies included in this review implies a possible overestimation of the effects of mind-body training, for which reason these results should be interpreted with caution. Finally, it is suggested that future authors employ more rigorous and solid methodological intervention protocols and standardized measurement instruments. They should also evaluate the effects of mind-body training in populations outside Asia.

## Figures and Tables

**Table 1:** Methodological quality of the articles included.

Article	1	2	3	4	5	6	7	8	9	10	11	Total
Hartmann et al. 2012 [25]	Y	Y	N	Y	N	N	N	Y	N	Y	Y	5
Koloverou et al. 2014 [26]	Y	Y	N	Y	N	N	Y	Y	N	Y	Y	6
Chen et al. 2020 [27]	Y	Y	N	Y	N	N	Y	Y	Y	Y	Y	7
Sarika et al. 2020 [28]	N	Y	N	N	N	N	N	Y	N	Y	Y	4
Singh & Khandelwal 2020 [29]	N	Y	Y	Y	N	N	N	Y	N	Y	Y	6
Ravari et al. 2020 [30]	Y	Y	Y	Y	N	N	N	Y	N	Y	Y	6
Chen et al. 2021 [31]	Y	Y	N	Y	N	N	N	Y	N	Y	Y	5
Yadav 2021 [32]	Y	Y	Y	Y	N	N	N	Y	N	Y	Y	6

Items: 1 = eligibility criteria; 2 = random allocation; 3 = concealed allocation; 4 = baseline comparability; 5 = blind subjects; 6 = blind therapists; 7 = blind assessors; 8 = adequate follow-up; 9 = intention-to-treat analysis; 10 = between-group comparisons; 11 = point estimates and variability; Y = Yes; N = No.

**Table 2:** Characteristics of the included studies.

Author and Year	Country	Sample CG/IG	Control Group	Intervention Group					
				Age	Intervention Type	Intervention Characteristics	Instruments and Variables Baseline Measures	Modifications over Time	
Hartmann et al. 2012 [25]	Germany	57/53	Usual care	CG: 59.30 ± 7.80 IG: 58.70 ± 7.40	Mindfulness-Based Stress-Reduction	Frequency: 1 session/week. Volume: 2 h. Duration: 8 weeks. Measurement points: Baseline, 8 weeks and 1 year.	PHQ Depression 6.40 ± 4.90 Stress 6.40 ± 3.60	8 weeks Depression 5.70 ± 0.53 Stress 4.90 ± 0.47	1 Year Depression 5.30 ± 0.48 * Stress 5.00 ± 0.42
Koloverou et al. 2014 [26]	Greece	28/25	Usual care	CG: 63.00 ± 8.00 IG: 60.52 ± 6.73	Relaxation breathing + progressive muscle relaxation Yoga-Based	Frequency: 2 sessions/day. Volume: 25 min. Duration: 8 weeks. Measurement points: Baseline and 8 weeks.	PSS 27.21 ± 8.02	8 weeks −2.6 ±1.10 *	-
Chen et al. 2020 [27]	Taiwan	60/60	Usual care	CG: 78.95 ± 7.12 IG: 78.85 ± 7.62	Mindfulness program	Frequency: 1 session/week. Volume: 1.5 h. Duration: 9 weeks. Measurement points: Baseline, 6 weeks and 9 weeks.	DASS-21 Depression 44.20 ± 16.57 RSS Relocated Stress 80.17 ± 12.77	6 weeks Depression 30.20 ± 11.89 Relocated Stress 67.91 ± 12.82	9 weeks Depression 23.17 ± 5.35 * Relocated Stress 54.85 ± 11.91 *
Sarika et al. 2020 [28]	India	15/15	Instructions on dietary modifications, exercises, and psychological counselling	CG: 48.53 ± 8.95 IG: 54.87 ± 10.27	Combination of yoga, pranayama, and meditation.	Frequency: 1 session/day, minimum 4 times per week. Volume: 28 min. Duration: 3 months. Measurement points: Baseline and 3 months.	PSS 21.13 ± 3.71	3 months 16.80 ± 3.34 *	-
Singh & Khandelwal 2020 [29]	India	115/112	General instruction booklet: diet prescription and general information of standard care.	CG:49.40 ± 8.70 IG: 50.30 ± 9.10	Yoga	Frequency: 1 session/day. Volume: Progressive increase in the duration of the different prescribed exercises, the full duration of the session is not specified. Duration: 3 months. Measurement points: Baseline and 3 months.	SSAI 37.5 ± 6.7 STAI 32.4 ± 6.5 total anxiety 69.9 ± 8.2 BDI 23.2 ± 4.3	3 months: SSAI 28.9 ± 10.2 * STAI 23.7 ± 6.0 * total anxiety 52.7 ± 12.1 * BDI 20.8 ± 3.2 *	-
Ravari et al. 2020 [30]	Iran	51/50	Routine care	CG:57.70 ± 5.65 IG: 56.40 ± 4.57	Mindfulness-based stress reduction	Frequency: 1 session/week. Volume: 2 h. Duration: 12 weeks. Measurement points: Baseline and 12 weeks.	DASS-21 Depression 17.15 ± 6.94 Anxiety 17.15 ± 7.33 Stress score 21.11 ± 8.23	12 weeks Depression 13.69 ± 8.07 * Anxiety 13.09 ± 7.67 * Stress score 17.36 ± 8.51 *	-
Chen et al. 2021 [31]	China	47/47	8-week-long intensive education program	CG:64.11 ± 4.36 IG: 63.98 ± 4.34	Mindfulness-based stress reduction + yoga	Frequency: 1 session/week Volume: not specified. Duration: 8 weeks. Measurement points: Baseline and 8 weeks.	SCL-90 Anxiety 1.94 ± 0.26 Depression 1.91 ± 0.25	8 weeks Anxiety 0.72 ± 0.17 * Depression 0.90 ± 0.21 *	-
Yadav 2021 [32]	India	50/50	Conventional treatment: Dietary and physical exercise counseling, oral hypoglycemic agents, and/or insulin as per their symptomatology	CG: 56.40 ± 11.72 IG: 52.24 ± 10.36	Deep breathing and Systematic Relaxation Yoga-Based + conventional treatment	Frequency: 1 session/week. Volume: 1 h. Duration: 6 months. Measurement points: Baseline and 6 months.	DASS-21 Depression 4.22 ± 2.49 Anxiety 6.90 ± 3.00 Stress 7.32 ± 2.59	6 Months Depression 3.24 ± 2.09 * Anxiety 5.79 ± 2.42 * Stress 6.01 ± 1.94 *	-

CG: Control Group; IG: Intervention Group; PHQ: Patient Health Questionnaire; PSS: Perceived stress scale; DASS-21: Depression, Anxiety, and Stress Scale-21; RSS: Relocation Stress Scale; SSAI: Spielberger’s State Anxiety Inventory; STAI = Spielberger’s Trait Anxiety Inventory; BDI: Beck Depression Inventory; SCL-90: Symptom Checklist 90. *: statistically significant (*p* < 0.05).

## Data Availability

All available data can be obtained by contacting the corresponding author.
